# Nationwide age-adjusted trends and inequities in vascular intestinal disorder mortality in the United States, 1999 to 2023

**DOI:** 10.1097/MD.0000000000048259

**Published:** 2026-04-10

**Authors:** Quanhuan Cui, Yonghui Liang

**Affiliations:** a Department of Emergency Medicine, Aerospace Center Hospital, Beijing, People’s Republic of China.

**Keywords:** CDC WONDER, mortality, United States, vascular intestinal disorder

## Abstract

Vascular intestinal disorders (VID), including acute mesenteric ischemia and related intestinal vascular insufficiency syndromes, are rare but highly lethal causes of abdominal emergency mortality. National trends in VID mortality, and whether improvements have been equitable across demographic and geographic groups, have not been characterized through 2023. We conducted a nationwide ecological analysis of VID mortality in the United States from 1999 to 2023 using the Centers for Disease Control and Prevention Wide-ranging Online Data for Epidemiologic Research. We included adults aged 25 years or older when VID was listed as the underlying cause of death. For each year, we extracted the national death count and the age-adjusted mortality rate (per 1,00,000 population) and evaluated temporal change. From 1999 to 2023, 2,03,870 VID deaths occurred. Annual deaths declined from 8711 in 1999 to 7676 in 2023. Over the same period, the national age-adjusted mortality rate decreased from 4.93 to 2.75/1,00,000, corresponding to an average annual percent change of −2.41%. Deaths were concentrated in older adults: individuals aged 75 to 84 years accounted for 66,509 deaths (32.6% of all VID deaths), and those aged 85 years or older accounted for 59,009 deaths (28.9%). Females accounted for 1,30,125 cumulative VID deaths and males for 73,745. Nonmetropolitan counties and Southern regions carried a disproportionate share of deaths. Although VID mortality declined on an age adjusted basis, the absolute burden remains greatest in the oldest adults and in structurally disadvantaged settings, indicating persistent geographic and demographic inequity during and beyond the COVID-19 era.

## 1. Introduction

VID comprises a spectrum of bowel ischemic and vascular pathologies. In the case of severe intestinal ischemia, patients may show signs of peritonitis, such as abdominal distension, protection and hypotension, which may be life-threatening.^[1,2]^ Despite improvements in multidetector computed tomography, endovascular revascularization, and modern critical care, VID remains among the most lethal abdominal emergencies in adults, with reported short term mortality frequently exceeding 59% in acute mesenteric ischemia.^[3]^ The incidence, severity, and fatality of these disorders increase steeply with advancing age, in part because older adults accumulate atherosclerotic disease, atrial fibrillation, heart failure, and other prothrombotic or low flow states, and are more likely to experience diagnostic delay or limited physiologic reserve.^[1,4]^ As a result, VID disproportionately affects older adults and continues to carry high in hospital and long term mortality even in contemporary series.^[5]^

Beyond age, structural features of acute care delivery strongly influence survival. Delays in vascular consultation or mesenteric revascularization are independently associated with higher short term mortality and catastrophic bowel loss in acute mesenteric ischemia.^[6]^ Access to rapid vascular and gastrointestinal surgical intervention, and timely transfer to tertiary centers, is not evenly distributed across US regions or between metropolitan and nonmetropolitan communities.^[6,7]^ VID related mortality also varies by sex, race and ethnicity, geography, and rurality. Recent national data suggest that women, several racial and ethnic minority populations, residents of the Midwest and South, and adults living outside large metropolitan areas bear a disproportionate share of VID deaths and show slower improvement in mortality over time.^[7]^

The COVID-19 pandemic further amplified this problem. The novel coronavirus can cause extensive vascular endothelial injury and hypercoagulability, leading to a high incidence of thrombotic complications. Therefore, it may also induce superior mesenteric artery thrombosis.^[8,9]^ Simultaneously, the pandemic period was characterized by saturation of intensive care capacity, reductions in operating room availability, staffing shortages, and delays in interhospital transfer, particularly during 2020 to 2021. These shocks plausibly altered both the burden and the distribution of VID related death in the United States.^[10,11]^

Although single center and regional reports have described the clinical course of specific VID subtypes such as acute mesenteric ischemia or colonic angiodysplasia, only limited work has quantified VID mortality at the national level.^[7]^ The most recent nationwide analysis before this study demonstrated persistent disparities by sex, race and ethnicity, rurality, and US. Census region, but covered mortality trends only through 2020. Important questions, therefore, remain: Has VID mortality continued to decline after 2020, including during and after successive COVID-19 waves; what is the trend in VID mortality across different types; which demographic groups continue to carry the greatest fatal burden; and have structural gaps between metropolitan and nonmetropolitan communities narrowed or widened?

Our study addresses these gaps by extending national VID mortality surveillance through 2023, assessing temporal trends in absolute deaths and age-adjusted mortality rates (AAMRs), and characterizing disparities by sex, race and ethnicity, age group, Census region, 2013 urbanization level, and VID subtype.

## 2. Materials and methods

### 2.1. Study design and data source

This study was a nationwide ecological analysis of mortality attributed to VID in the United States from 1999 through 2023. The analytic population included US resident decedents aged 25 years or older.

The mortality data were obtained from the Centers for Disease Control and Prevention Wide-ranging Online Data for Epidemiologic Research (CDC WONDER) Underlying Cause of Death database.^[12,13]^ This database is compiled by the National Center for Health Statistics from state-issued death certificates and aggregated into the National Vital Statistics System. For each calendar year, we extracted the number of VID deaths, the crude mortality rate per 1,00,000 population, and the AAMR per 1,00,000 population. AAMR is calculated in CDC WONDER using direct standardization to the year 2000 US standard population and is provided with standard errors and 95% confidence intervals.^[14]^

### 2.2. Measures and stratification

The primary outcomes were: the annual number of VID deaths; and the VID AAMR per 1,00,000 population. Deaths were included when VID ICD-10 codes were recorded as the underlying cause of death. Multiple-cause mentions were not used in the primary analyses. Because CDC WONDER does not generate AAMRs for each 10-year age band, crude mortality rates per 1,00,000 population with 95% confidence intervals were used to summarize mortality in specific age groups.

VID mortality was examined overall and within strata defined by sex (female, male), 10-year age group (25–34, 35–44, 45–54, 55–64, 65–74, 75–84, and ≥85 years), race and ethnicity (Hispanic, non-Hispanic (NH) White, NH Black, and NH Other), US Census region (Northeast, Midwest, South, West), 2013 urbanization classification, and VID subtype. In CDC WONDER, the NH Other category aggregates American Indian or Alaska Native, Asian, Native Hawaiian or Other Pacific Islander, and multiracial individuals, consistent with national vital statistics practice.^[15,16]^ Race and ethnicity analyses were restricted to decedents with non-missing race/ethnicity information. Records with unknown or unclassified race/ethnicity were not included in race-stratified totals. VID subtypes included acute vascular disorders of intestine (ICD-10 code K55.0), chronic vascular disorders of intestine (ICD-10 code K55.1), angiodysplasia of colon (ICD-10 code K55.2), and other VID (ICD-10 code K55.8–55.9). The category “other vascular disorders of intestine” (K55.8, K55.9) is clinically heterogeneous and may include unspecified intestinal vascular insufficiency and other ischemic bowel presentations that are not further subclassified in vital-statistics coding.

County of residence for each decedent was linked to the 2013 National Center for Health Statistics Urban–Rural Classification Scheme for Counties, which distinguishes metropolitan and nonmetropolitan areas in federal health surveillance.^[17,18]^ In this classification, AAMRs by urbanization are available through 2020. Therefore, for metropolitan and nonmetropolitan strata, values labeled “2023 AAMR” reflect the most recent available AAMR, which corresponds to data through 2020 rather than true 2023 rates.

### 2.3. Statistical analysis

We summarized cumulative VID deaths from 1999 to 2023 overall and within each stratum. For each stratum we also calculated the percentage change in annual VID deaths between 1999 and 2023 using the expression: (deaths in 2023 minus deaths in 1999) divided by deaths in 1999, multiplied by 100%.

Temporal trends in VID mortality were modeled using Joinpoint regression. Joinpoint regression fits segmented log linear models to annual mortality rates, estimates the annual percent change (APC) for each segment, and uses permutation testing to determine whether a change in slope is statistically significant.^[19]^ The model yields the average annual percent change (AAPC) with 95% CI, which summarizes the overall yearly change in the mortality rate across the full study interval on a log scale.^[19,20]^ And APC was defined as the average yearly percent change within each segment, and AAPC as a length-weighted summary of APCs across the entire study period. Joinpoint models were fitted to annual mortality rates with a maximum of 6 joinpoints for each 25-year time series. Model selection was automated using permutation test with an overall significance level of .05. Serial autocorrelation was not explicitly modeled in the primary joinpoint analysis.

Joinpoint analyses were performed for national AAMR and for AAMR stratified by sex, race and ethnicity, Census region, and VID subtype for 1999 through 2023. For age groups, crude mortality rates per 1,00,000 were modeled instead of AAMR because AAMR is not generated for each 10-year age band in CDC WONDER. For the 2013 urbanization classification (metropolitan vs nonmetropolitan), the Joinpoint analysis covered 1999 through 2020, the most recent period for which age-adjusted rates are available by that stratification. All data extraction, quality checks, and figure generation were conducted in R (version 4.4.3). Because all data were publicly available and de-identified, institutional review board approval and informed consent were not required.

### 2.4. Ethical considerations

This study used publicly accessible, de-identified aggregate data. The data from CDC WONDER adheres to federal confidentiality standards and contains no individual identifiers. As such, the analysis was exempt from Institutional Review Board review and did not require informed consent.

## 3. Results

### 3.1. Overall mortality trends

**Figure 1.**
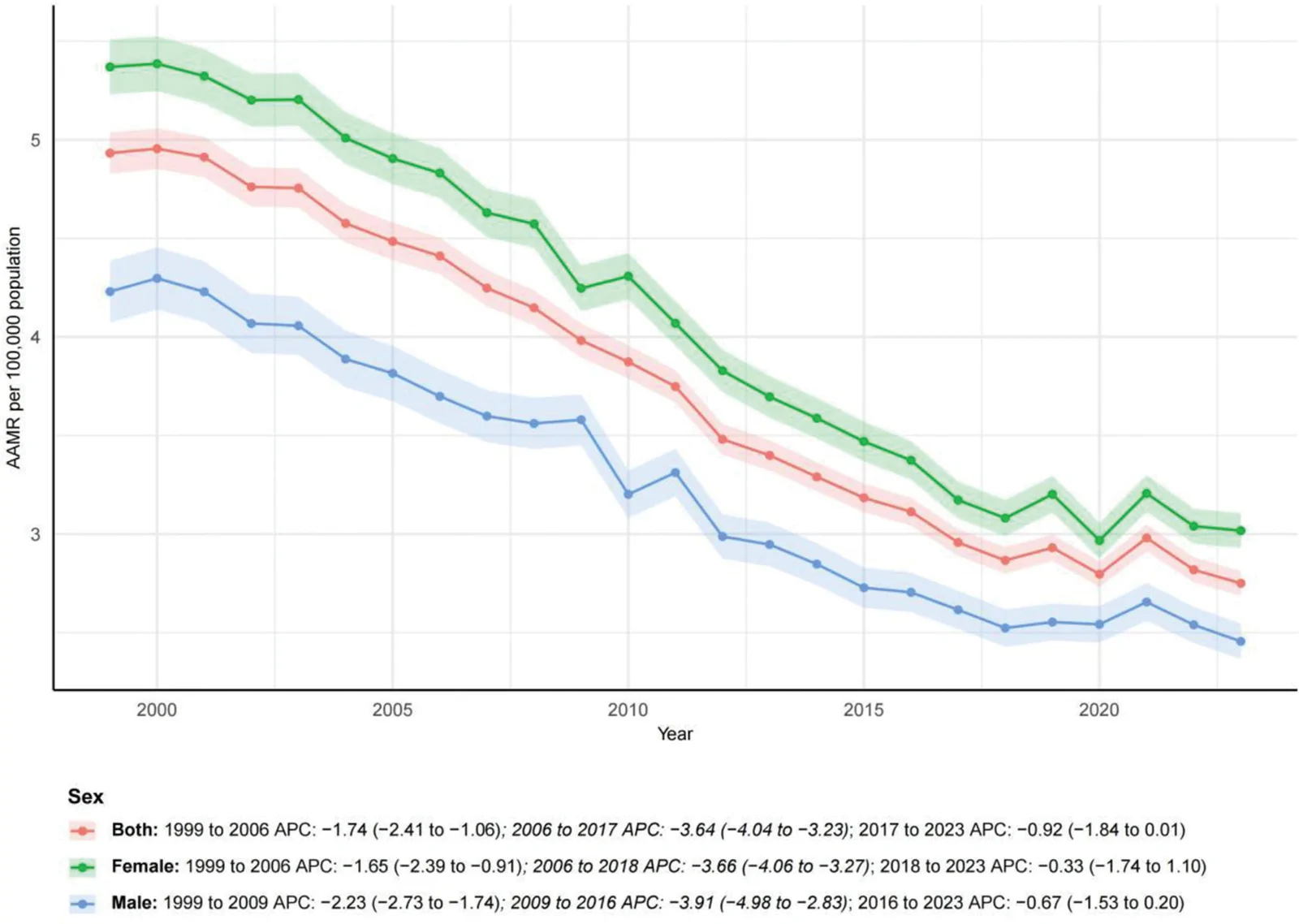
Temporal trends in AAMRs for vascular intestinal disorder in the United States, overall and by sex, 1999 to 2023. AAMR = age-adjusted mortality rate, APC = annual percent change.

From 1999 to 2023, the United States recorded 2,03,870 deaths with VID as the underlying cause of death. In absolute terms, annual deaths decreased from 8711 in 1999 to 7676 in 2023, a change of −11.88%. In rate terms, the national AAMR declined from 4.93 to 2.75/1,00,000, with an AAPC of −2.41% (Fig. 1; Table 1).

**Table 1:** Vascular intestinal disorder deaths and AAMR in the United States in 1999 and 2023, and their temporal trends.

Characteristic	Deaths				AAMR		
	1999–2023	1999	2023	Percent change	1999 (95% CI)	2023 (95% CI)	AAPC (95% CI)
Both	2,03,870	8711	7676	−11.88	4.93 (4.83–5.04)	2.75 (2.69–2.81)	−2.41 (−2.74 to −2.08)
Female	1,30,125	5803	4627	−20.27	5.37 (5.23–5.51)	3.02 (2.93–3.11)	−2.39 (−2.77 to −2.01)
Male	73,745	2908	3049	4.85	4.23 (4.07–4.39)	2.46 (2.37–2.54)	−2.27 (−2.69 to −1.85)
Census region							
Northeast	38,754	1734	1403	−19.09	4.60 (4.39–4.82)	2.73 (2.58–2.87)	−2.48 (−2.67 to −2.30)
Midwest	49,404	2155	1723	−20.05	5.10 (4.88–5.31)	2.97 (2.83–3.11)	−2.21 (−2.71 to −1.71)
South	73,783	3143	2901	−7.7	5.09 (4.92–5.27)	2.73 (2.62–2.83)	−2.38 (−2.82 to −1.94)
West	41,929	1679	1649	−1.79	4.86 (4.63–5.10)	2.66 (2.53–2.79)	−2.67 (−3.25 to −2.09)
Race							
Hispanic	11,857	317	654	106.31	3.90 (3.45–4.35)	2.26 (2.08–2.44)	−2.59 (−3.25 to −1.93)
NH Black	20,376	843	901	6.88	5.77 (5.37–6.16)	3.32 (3.10–3.54)	−2.14 (−2.55 to −1.72)
NH White	165,946	7408	5796	−21.76	4.96 (4.85–5.08)	2.87 (2.79–2.95)	−2.24 (−2.58 to −1.90)
NH Other	5219	124	300	141.94	2.77 (2.26–3.28)	1.55 (1.37–1.73)	−3.16 (−3.56 to −2.75)
2013 urbanization level*							
Metropolitan	1,63,824	6995	6190	−11.51	4.92 (4.80–5.03)	2.62 (2.56–2.69)	−3.12 (−3.43 to −2.81)
Nonmetropolitan	40,046	1716	1486	−13.40	5.11 (4.87–5.35)	3.72 (3.53–3.90)	−1.38 (−2.07 to −0.68)
Age group†							
25–34	839	35	58	65.71	0.09 (0.06–0.12)	0.13 (0.10–0.16)	2.46 (1.40–3.53)
35–44	2488	93	142	52.69	0.21 (0.17–0.25)	0.32 (0.27–0.37)	2.44 (0.73–4.17)
45–54	8530	291	339	16.49	0.80 (0.70–0.89)	0.84 (0.75–0.93)	0.61 (−0.10 to 1.33)
55–64	22,396	748	1030	37.7	3.15 (2.92–3.37)	2.46 (2.31–2.61)	−0.85 (−1.95 to 0.26)
65–74	44,099	1850	2045	10.54	10.04 (9.59–10.50)	5.90 (5.64–6.15)	−2.26 (−2.59 to −1.94)
75–84	66,509	3120	2376	−23.85	25.52 (24.63–26.42)	12.94 (12.42–13.46)	−2.90 (−3.26 to −2.53)
85+	59,009	2574	1686	−34.5	61.96 (59.57–64.36)	27.22 (25.92–28.51)	−3.68 (−4.15 to −3.20)

AAMR = age-adjusted mortality rate, AAPC = average annual percent change, CI = confidence interval, NH = non-Hispanic.

* AAMR in 2023 is based on data in 2020.

† AAMR was replaced by crude mortality.

**Figure 2.**
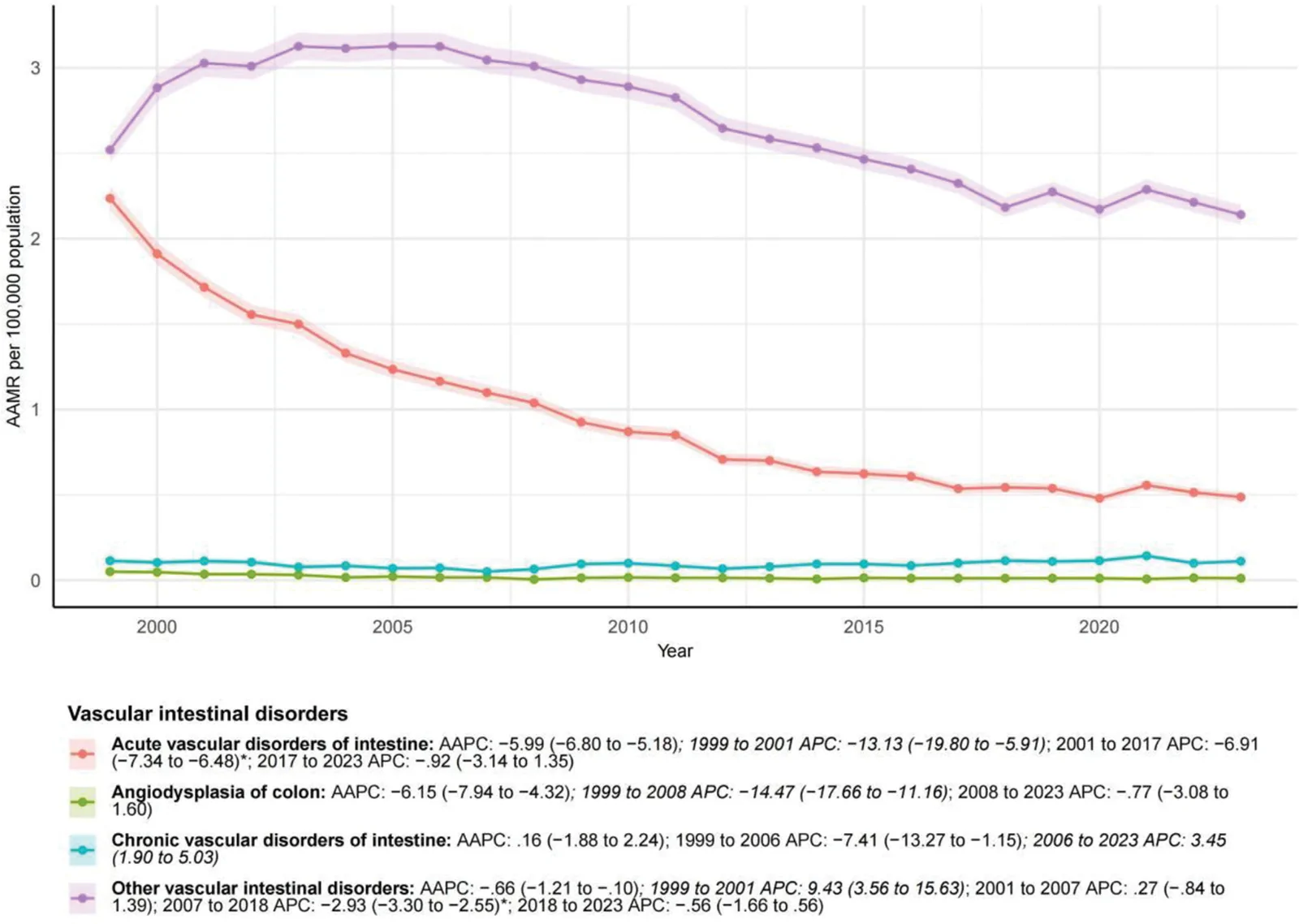
Temporal trends in AAMRs for vascular intestinal disorder subtypes, 1999 to 2023. AAMR = age-adjusted mortality rate, AAPC = average annual percent change, APC = annual percent change,.

Across subtypes, other VID contributed most deaths (1,46,313, 71.8%), followed by acute vascular disorders of the intestine (50,861, 24.9%), chronic vascular disorders of the intestine (5579, 2.7%), and angiodysplasia of the colon (1117, 0.5%; Table 2). Joinpoint analysis showed an overall decline in VID AAMR over time (Fig. 2).

**Table 2:** Death of various vascular intestinal disorders and AAMR in the United States in 1999 and 2023 and their time trends.

Characteristic	Deaths				AAMR		
	1999–2023	1999	2023	Percent change	1999 (95% CI)	2023 (95% CI)	AAPC (95% CI)
Acute vascular disorders of intestine	50,861	3979	1395	−64.94	2.24 (2.17, 2.31)	0.49 (0.46, 0.51)	−5.99 (−6.80 to −5.18)
Chronic vascular disorders of intestine	5579	210	336	60.00	0.11 (0.10, 0.13)	0.11 (0.10, 0.12)	0.16 (−1.88 to 2.24)
Angiodysplasia of colon	1117	90	42	−53.33	0.05 (0.04, 0.06)	0.01 (0.01, 0.02)	−6.15 (−7.94 to −4.32)
Other vascular intestinal disorders	146,313	4432	5903	33.19	2.52 (2.45, 2.59)	2.14 (2.08, 2.20)	−0.66 (−1.21 to −0.10)

AAMR = age-adjusted mortality rate, AAPC = average annual percent change, CI = confidence interval.

### 3.2. Mortality by subtype

Subtype patterns differed when absolute burden and rate-based trends were considered together (Table 2; Fig. 2). Acute vascular disorders of the intestine showed the largest decline, with annual deaths decreasing from 3979 to 1395 (−64.94%) and AAMR decreasing from 2.24 to 0.49/1,00,000 (AAPC −5.99%).

For other VID, annual deaths increased from 4432 to 5903 (+33.19%), whereas AAMR decreased from 2.52 to 2.14/1,00,000 (AAPC −0.66%). This divergence between counts and rates should be interpreted cautiously, because this category is clinically heterogeneous and may be influenced by coding shifts and diagnostic substitution.

Chronic vascular disorders of the intestine showed increasing annual deaths (210–336, +60.00%) with little change in AAMR (0.11–0.11/1,00,000; AAPC 0.16%). Angiodysplasia of the colon declined in both annual deaths (90–42, −53.33%) and AAMR (0.05–0.01/1,00,000; AAPC −6.15%).

### 3.3. Trends by sex

From 1999 to 2023, cumulative VID deaths were higher in females than in males (1,30,125 vs 73,745; Table 1; Fig. 1). In absolute terms, annual deaths declined in females (5803–4627, −20.27%) but were slightly higher in males in 2023 than in 1999 (2908–3049, +4.85%).

In rate terms, AAMR declined in both sexes. Female AAMR decreased from 5.37 to 3.02/1,00,000 (AAPC −2.39%), and male AAMR decreased from 4.23 to 2.46/1,00,000 (AAPC −2.27%).

### 3.4. Trends by age group

**Figure 3.**
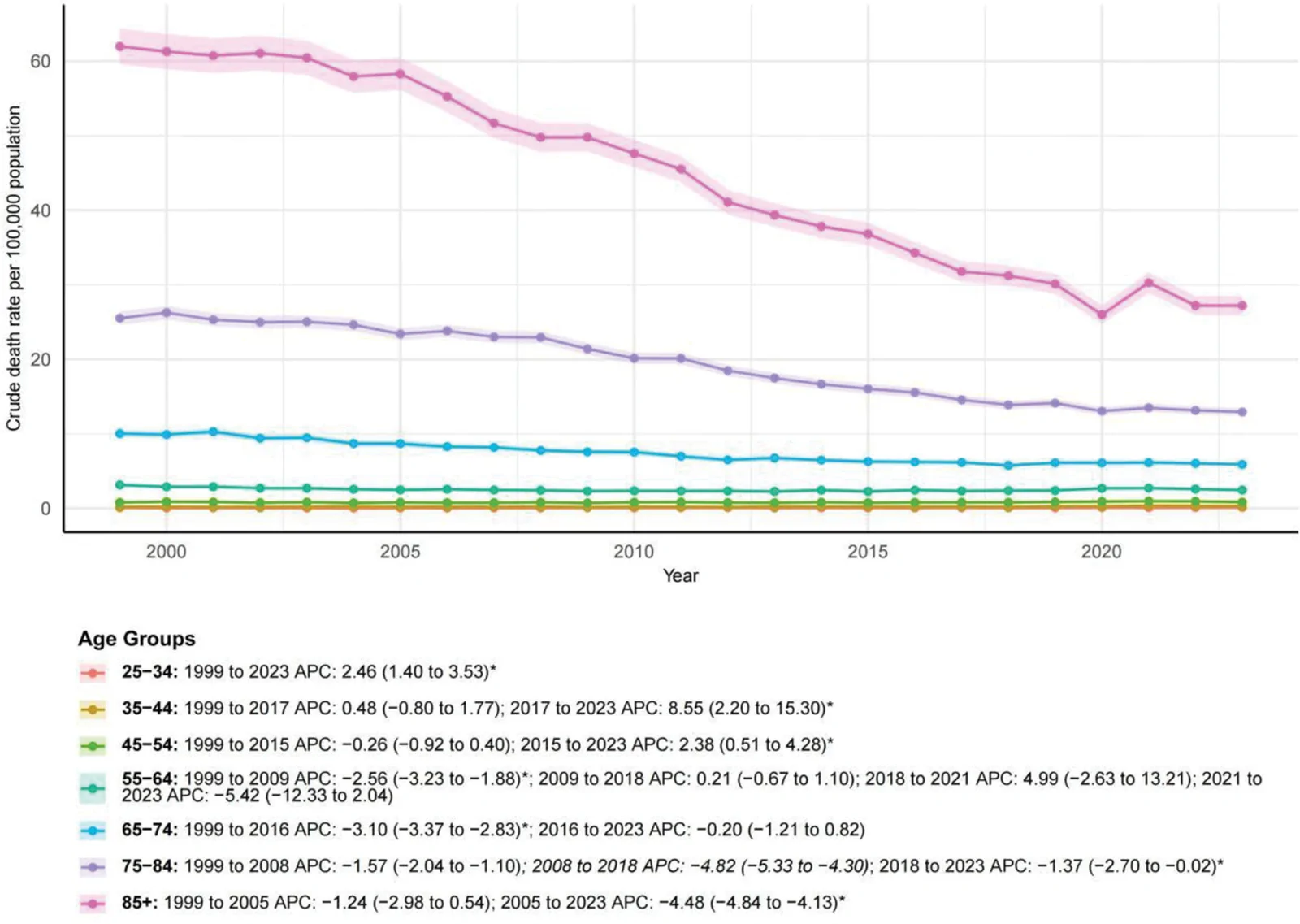
Temporal trends in crude death rates for vascular intestinal disorder by age group, 1999 to 2023. APC = annual percent change.

VID mortality burden was concentrated in older adults (Table 1; Fig. 3). Individuals aged 75 to 84 years accounted for 66,509 deaths (32.6%), those aged 85 years or older for 59,009 deaths (28.9%), and those aged 65 to 74 years for 44,099 deaths (21.6%). Together, these 3 groups represented more than 80% of all VID deaths.

Age gradients were substantial in crude mortality rates. In 2023, the crude mortality rate was 27.22/1,00,000 in adults aged 85 years or older, 5.90 in adults aged 65 to 74 years, and 0.13 in adults aged 25 to 34 years. Over time, crude rates declined in older groups: 65 to 74 years (10.04–5.90; AAPC −2.26%), 75 to 84 years (25.52–12.94; AAPC −2.90%), and 85 years or older (61.96–27.22; AAPC −3.68%).

### 3.5. Trends by race and ethnicity

**Figure 4.**
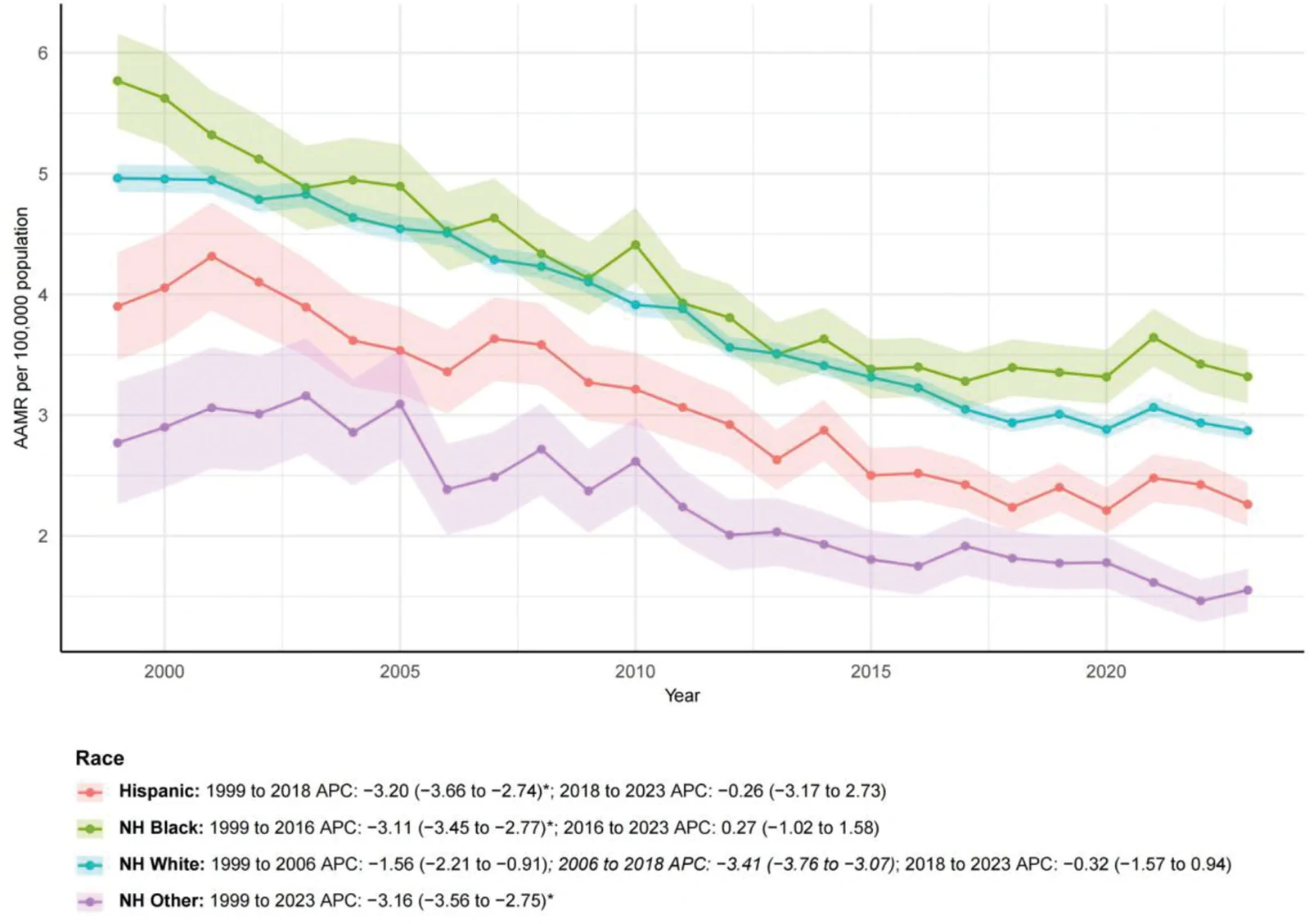
Temporal trends in AAMRs for vascular intestinal disorder by race/ethnicity, 1999 to 2023. AAMR = age-adjusted mortality rate, APC = annual percent change, NH = non-Hispanic.

From 1999 to 2023, cumulative VID deaths were 1,65,946 in NH White individuals, 20,376 in NH Black individuals, 11,857 in Hispanic individuals, and 5219 in NH Other individuals (Table 1; Fig. 4). Annual deaths decreased in NH White individuals (7408–s5796, −21.76%) but increased in NH Black (843–901, +6.88%), Hispanic (317–654, +106.31%), and NH Other groups (124–300, +141.94%).

Despite these count increases in several groups, AAMR declined in all groups. AAMR declined from 5.77 to 3.32 in NH Black individuals (AAPC −2.14%), from 4.96 to 2.87 in NH White individuals (AAPC −2.24%), from 3.90 to 2.26 in Hispanic individuals (AAPC −2.59%), and from 2.77 to 1.55 in NH Other individuals (AAPC −3.16%). In 2023, NH Black individuals still had the highest AAMR (3.32/1,00,000).

### 3.6. Trends by census region

**Figure 5.**
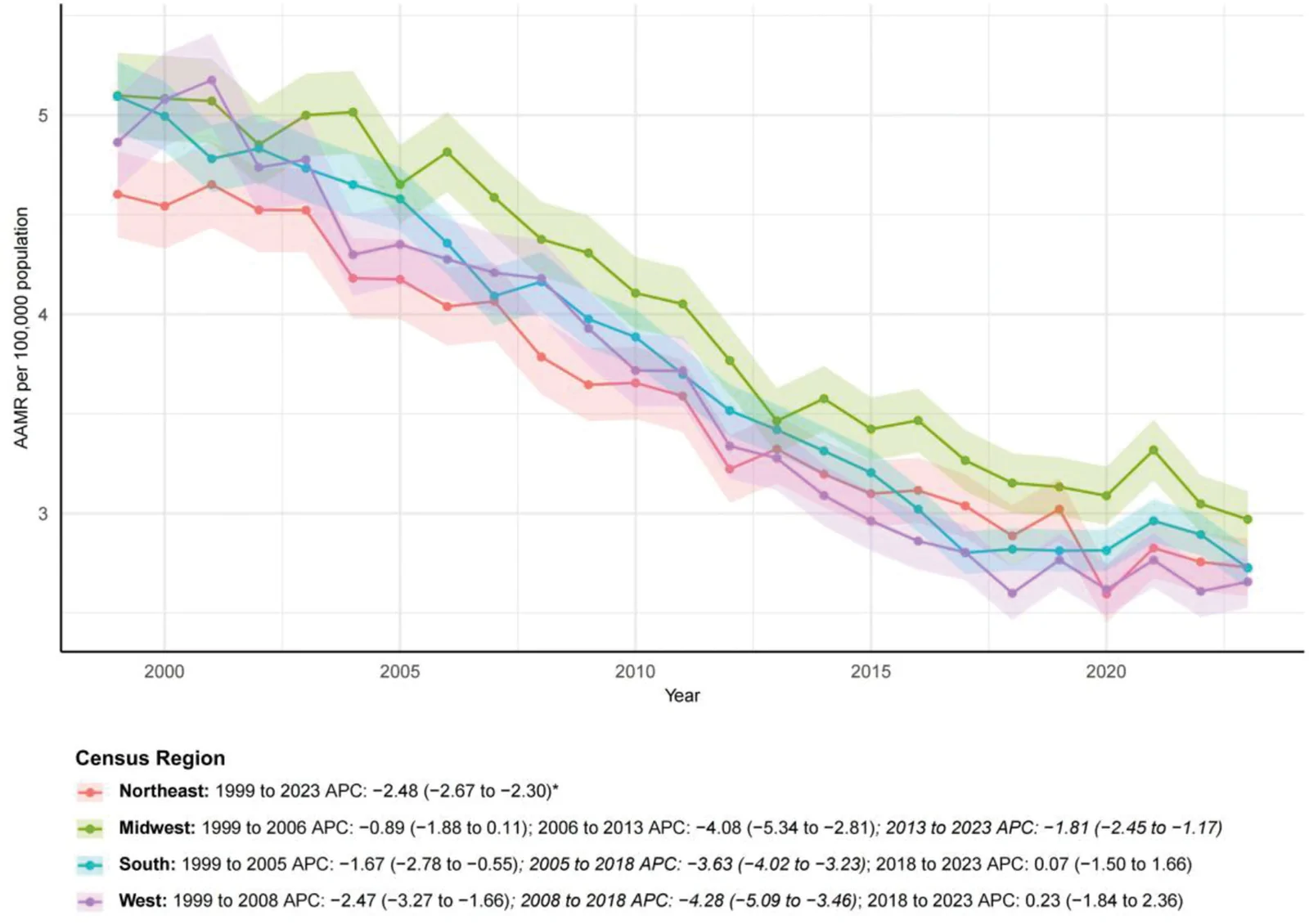
Temporal trends in AAMRs for vascular intestinal disorder by United States census region (Northeast, Midwest, South, West), 1999 to 2023. AAMR = age-adjusted mortality rate, APC = annual percent change.

Across census regions, cumulative VID deaths were highest in the South (73,783; 36.2%), followed by the Midwest (49,404), West (41,929), and Northeast (38,754; Table 1; Fig. 5).

AAMRs declined in every region. In 1999, regional AAMRs ranged from 4.60 to 5.10/1,00,000. By 2023, they ranged from 2.66 to 2.97/1,00,000. AAPCs were −2.21% in the Midwest, −2.38% in the South, −2.48% in the Northeast, and −2.67% in the West.

### 3.7. Trends by urbanization level

**Figure 6.**
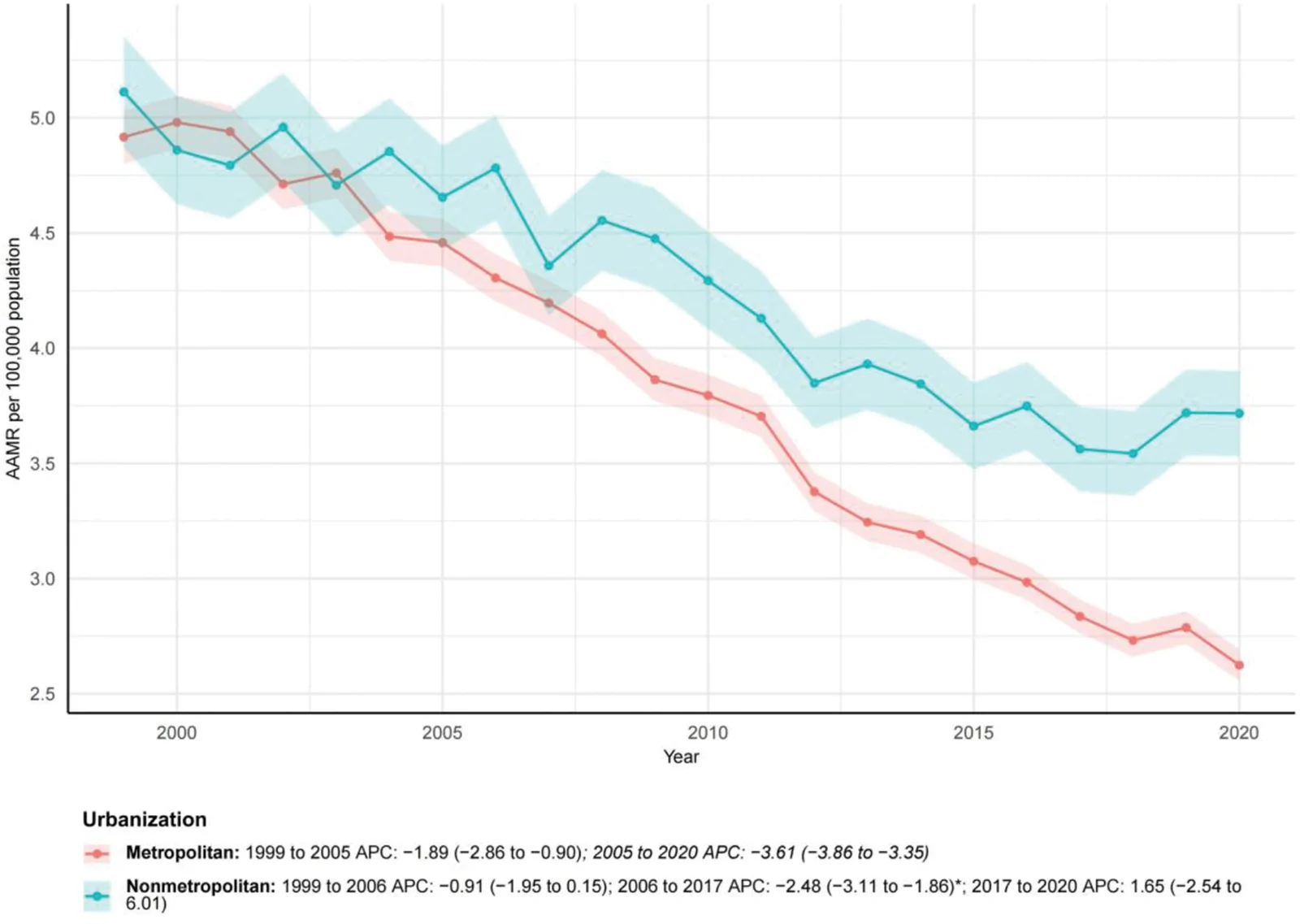
Temporal trends in AAMRs for vascular intestinal disorder by urbanization level, 1999 to 2020. AAMR = age-adjusted mortality rate, APC = annual percent change.

Using the 2013 NCHS urban–rural classification, metropolitan counties accounted for 1,63,824 deaths (80.4%) and nonmetropolitan counties for 40,046 deaths (19.6%) during 1999 to 2023 (Table 1; Fig. 6).

Annual deaths declined in both settings, from 6995 to 6190 in metropolitan counties (−11.51%) and from 1716 to 1486 in nonmetropolitan counties (−13.40%). AAMRs also declined in both settings, from 4.92 to 2.62/1,00,000 in metropolitan counties (AAPC −3.12%) and from 5.11 to 3.72 in nonmetropolitan counties (AAPC −1.38%). The nonmetropolitan to metropolitan disparity widened over time. The AAMR difference increased from 0.19/1,00,000 in the earliest year to 1.10 in the most recent year available for this urbanization-specific rate series (through 2020), corresponding to a rate ratio of 1.42.

## 4. Discussion

The present nationwide analysis shows that VID mortality in the United States declined substantially from 1999 to 2023 when assessed using AAMR, and the AAPC of VID mortality was strongly negative across the study interval, which is consistent with reports that outcomes in VID have improved over the last 2 decades in parallel with advances in diagnosis and reperfusion strategy.^[1,21,22]^ We also find that the burden of the elderly, rural counties and NH black deceased is disproportionate, which not only has the highest mortality burden, but also improves more slowly. Current international guidelines define acute mesenteric ischemia as an abrupt interruption of mesenteric blood flow that rapidly produces intestinal ischemia, systemic sepsis, and multiorgan failure, and they indicate that improved survival is associated with the timely use of contrast enhanced computed tomography, prompt broad spectrum antibiotics and systemic anticoagulation, aggressive resuscitation, and urgent mesenteric revascularization or resection of necrotic bowel when indicated.^[4]^ At the same time, the guidelines also recommend revascularization for patients with chronic mesenteric ischemia to relieve their chief symptoms and improve overall quality of life.^[23]^ In short, with the continuous advancement of diagnostic and therapeutic techniques, ongoing advances in diagnosis and treatment are associated with the long term decline in VID age adjusted mortality observed in this study.

Our data indicate that deaths from VID are concentrated in the elderly population, particularly among those aged 75 years and above. This is consistent with evidence from clinical and population-based studies: the incidence of mesenteric ischemia rises sharply with age, and mortality is also concentrated in the elderly. Potential contributing factors include advanced atherosclerotic vascular disease, arrhythmia, physical frailty, and delayed medical consultation.^[4,5,24]^

We also found that over the entire study period, the cumulative number of VID deaths was higher in females than in males. This may be attributed to the following: premenopausal women face an elevated risk of vascular thromboembolic diseases due to the use of hormonal contraceptives; women generally have a longer life expectancy than men, increasing their likelihood of developing chronic vascular diseases, especially after menopause; and postmenopausal hormone replacement therapy also raises the risk of ischemic diseases.^[7,25]^ However, the gender gap in VID deaths has narrowed in recent years, and the number of VID deaths in males has not decreased correspondingly. This suggests the existence of gender-related differences in patients’ clinical manifestations, surgical strategies, and survival outcomes. During the COVID-19 pandemic, mortality rates (for VID) surged significantly in both males and females, which was closely associated with strained healthcare resources and a decline in emergency surgical volumes during this period.^[10,26]^

We observed significant racial and geographic disparities in VID mortality rates. In recent years, NH Black individuals in this study have consistently borne a disproportionately higher mortality burden than other populations. This aligns with evidence from a nationwide cohort study on acute mesenteric ischemia, which showed that African American patients have historically had higher in-hospital complication and mortality rates compared to White patients, and are also more likely to undergo bowel resection without revascularization.^[27]^ This pattern may reflect delays in hospital presentation or diagnosis, which may contribute to a higher likelihood of intestinal necrosis requiring resection. Therefore, for patients with time-sensitive surgical emergencies requiring urgent intervention, structural barriers such as limited access to advanced vascular and endovascular treatments, delayed transfer, and insufficient critical care resources are associated with poorer outcomes for racial minority patients.

We also documented that the South contributed the largest share of VID deaths and that nonmetropolitan counties had persistently higher mortality rates than metropolitan counties, which is consistent with national work demonstrating that rural hospitals have poorer spatial access to emergency general surgery and complex vascular intervention than urban centers and that distance and transfer delay are strongly linked to survival in abdominal catastrophes.^[28-30]^ High poverty rural counties in the Southern United States have the highest cardiovascular mortality rates in the nation and the largest mortality gap relative to wealthy urban counties, which signals overlapping regional concentration of vascular risk and limited specialty capacity that likely extends to mesenteric and intestinal ischemia.^[28,29]^

These findings have direct clinical and public health implications. First, the combination of declining national VID mortality and persistent gradients by age, sex, race, region, and rurality supports the view that further reductions are achievable if best practices become equitably available. Multiple contemporary guidelines and multicenter consortia emphasize rapid computed tomography angiography based triage, an endovascular first or hybrid revascularization strategy when feasible, and coordinated critical care after reperfusion as modifiable drivers of survival in acute mesenteric ischemia. Our results reinforce calls to treat time to vascular imaging, time to mesenteric reperfusion, and timely access to advanced postoperative critical care as quality indicators that can be tracked across regions and health systems, including rural and safety net hospitals. Second, because VID mortality remains concentrated in the oldest adults, in rural high poverty areas, and in racially minoritized groups, equity focused deployment of advanced vascular, endovascular, and critical care capabilities should be considered a national priority in order to prevent avoidable intestinal ischemic deaths.

Several limitations should inform interpretation. VID is not a single disease entity; it encompasses various types, including acute and chronic intestinal vascular disorders. The underlying cause of death documented on death certificates is jointly determined by clinical judgment, imaging studies, operative findings, and disease coding practices, all of which have evolved over the 25-year period. Consequently, some misclassification across VID subtypes may occur. The CDC Wonder does not provide AAMR for specific 10-year age groups. As a result, the analysis of age-specific mortality trends can only rely on crude mortality rates, which fail to account for the impact of population aging. As a death-certificate based dataset, CDC WONDER does not provide detailed clinical severity, comorbidity burden, treatment pathways, or time-to-intervention variables, which limits mechanistic interpretation. Lastly, AAMR stratified by urbanization category is currently only updated through 2020, forcing us to interpret the 2020 data as the most recent mortality levels for metropolitan and nonmetropolitan areas.

Despite these limitations, the overall pattern still indicates that VID remains clinically severe and unequally distributed across populations. We observed a national decline in AAMR, a high concentration of deaths among older adults, and persistent disparities by race and ethnicity, region, rurality, and sex. In younger age groups, some relative increases were observed; however, absolute mortality rates remained low, and year to year fluctuations may be sensitive to small counts.

## 5. Conclusion

From 1999 to 2023, age-adjusted VID mortality in the United States declined, but absolute mortality burden remained substantial and concentrated in older adults. Persistent disparities by race and ethnicity, region, and urbanization indicate uneven population benefit from advances in diagnosis and care. These findings support equity-focused improvements in timely diagnosis, transfer, and access to high-capability acute care.

## Acknowledgments

We express our gratitude to the Core facility and Bioinformatics Laboratory of The First Hospital of Jilin University for the training and generous sharing of experiences and codes.

## Author contributions

**Conceptualization:** Yonghui Liang.

**Data curation:** Quanhuan Cui.

**Formal analysis:** Quanhuan Cui.

**Funding acquisition:** Yonghui Liang.

**Investigation:** Quanhuan Cui, Yonghui Liang.

**Methodology:** Quanhuan Cui.

**Project administration:** Yonghui Liang.

**Resources:** Quanhuan Cui.

**Software:** Yonghui Liang.

**Supervision:** Yonghui Liang.

**Validation:** Quanhuan Cui, Yonghui Liang.

**Visualization:** Quanhuan Cui, Yonghui Liang.

**Writing – original draft:** Quanhuan Cui, Yonghui Liang.

**Writing – review & editing:** Quanhuan Cui, Yonghui Liang.
